# Application of Hyperspectral Imaging for Maturity and Soluble Solids Content Determination of Strawberry With Deep Learning Approaches

**DOI:** 10.3389/fpls.2021.736334

**Published:** 2021-09-10

**Authors:** Zhenzhu Su, Chu Zhang, Tianying Yan, Jianan Zhu, Yulan Zeng, Xuanjun Lu, Pan Gao, Lei Feng, Linhai He, Lihui Fan

**Affiliations:** ^1^Institute of Biotechnology, Zhejiang University, Hangzhou, China; ^2^School of Information Engineering, Huzhou University, Huzhou, China; ^3^College of Information Science and Technology, Shihezi University, Shihezi, China; ^4^Key Laboratory of Oasis Ecology Agriculture, Shihezi University, Shihezi, China; ^5^College of Biosystems Engineering and Food Science, Zhejiang University, Hangzhou, China; ^6^Key Laboratory of Spectroscopy Sensing, Ministry of Agriculture and Rural Affairs, Hangzhou, China; ^7^Hangzhou Liangzhu Linhai Vegetable and Fruit Professional Cooperative, Hangzhou, China

**Keywords:** hyperspectral imaging, strawberry, saliency map, ResNet, soluble solids content

## Abstract

Maturity degree and quality evaluation are important for strawberry harvest, trade, and consumption. Deep learning has been an efficient artificial intelligence tool for food and agro-products. Hyperspectral imaging coupled with deep learning was applied to determine the maturity degree and soluble solids content (SSC) of strawberries with four maturity degrees. Hyperspectral image of each strawberry was obtained and preprocessed, and the spectra were extracted from the images. One-dimension residual neural network (1D ResNet) and three-dimension (3D) ResNet were built using 1D spectra and 3D hyperspectral image as inputs for maturity degree evaluation. Good performances were obtained for maturity identification, with the classification accuracy over 84% for both 1D ResNet and 3D ResNet. The corresponding saliency maps showed that the pigments related wavelengths and image regions contributed more to the maturity identification. For SSC determination, 1D ResNet model was also built, with the determination of coefficient (*R*^2^) over 0.55 of the training, validation, and testing sets. The saliency maps of 1D ResNet for the SSC determination were also explored. The overall results showed that deep learning could be used to identify strawberry maturity degree and determine SSC. More efforts were needed to explore the use of 3D deep learning methods for the SSC determination. The close results of 1D ResNet and 3D ResNet for classification indicated that more samples might be used to improve the performances of 3D ResNet. The results in this study would help to develop 1D and 3D deep learning models for fruit quality inspection and other researches using hyperspectral imaging, providing efficient analysis approaches of fruit quality inspection using hyperspectral imaging.

## Introduction

Strawberry, a kind of fruit cultivated worldwide, is favored by consumers due to the unique characteristics, such as characteristic aroma, sweetness, and rich in nutrition. Strawberries are commonly eaten fresh, and they are sources and ingredients of the other foods. Maturity is an important quality index of fruits, related to eating quality, harvest, storage, and trade. It has short shelf-life, and strawberries with different maturity degree have different shelf-life. Fully matured strawberries have the shortest shelf-life (Rahman et al., 2014). Immature strawberries can become mature and over-mature quickly. They are vulnerable to physical damage, especially for the matured ones (Aliasgarian et al., 2015). Damaged strawberries will become rotten. Generally, nearly-mature strawberries are harvested and stored for trade. Exploring the appropriate maturity degrees for harvest is of importance for the growth management, storage, and trade.

As a matter of fact, it is easy for consumers to evaluate the maturity of the strawberry by observing its color, which is labor-cost and low-efficiency. Internal quality is another aspect that consumers care about for strawberry, which is more difficult to be estimated by observing the fruits. Researchers, planters, and traders have tried to develop non-destructive and automatic systems for the maturity and quality monitoring of strawberry. Computer vision has been proved to be quite efficient for the strawberry maturity determination, and it lacks the ability to determine the internal quality (Xu and Zhang, 2007; He et al., 2017; Oo and Aung, 2018). Near-infrared spectroscopy is a technique that can determine internal quality of strawberries (Shen et al., 2018; Mancini et al., 2020). However, near-infrared spectroscopy lacks in spatial information of the samples.

By integrating the computer vision and spectroscopy techniques, hyperspectral imaging has been utilized as a non-destructive and rapid analytical technique in various fields, such as nanoscale materials (Dong et al., 2018), plant seeds (Feng et al., 2019a), agricultural and food products (Jia et al., 2020), horticultural products (Lu et al., 2020), biological tissues (Rehman and Qureshi, 2020), wound care (Saiko et al., 2020), and fruits damage (He et al., 2021). Hyperspectral imaging has also been proved as an effective analytical technique for the fruit quality and safety inspection. Hussain et al. (2018) reviewed the use of hyperspectral imaging for fruit ripening and maturity. Lu et al. (2020) reviewed the use of hyperspectral imaging for fruit color, physiological disorders, damages, maturity, etc. He et al. (2021) reviewed the use of hyperspectral imaging for fruits damage inspection. Moreover, researchers have adopted hyperspectral imaging in strawberry for maturity and quality determination. Zhang et al. (2016) used hyperspectral imaging to evaluate the ripeness of strawberry. Elmasry et al. (2007) used hyperspectral imaging to determine the moisture content, total soluble solids, and acidity in strawberry. Liu et al. (2018) used hyperspectral imaging to identify bruise and fungi contamination in strawberries. Siedliska et al. (2018) used hyperspectral imaging to detect fungal infections in strawberry. Liu et al. (2019) used hyperspectral imaging to detect the decay of postharvest strawberry. Shao et al. (2020) used hyperspectral imaging to evaluate the strawberry ripeness. Weng et al. (2020) used hyperspectral imaging to determine the soluble solid content (SSC), pH, and vitamin C in strawberry. Hyperspectral images can provide spectral and image information. Spectral information can be used to determine the internal quality, and image information can be used to determine the external quality. Based on the information extracted from hyperspectral images, calibration models can be built to determine the external and internal quality.

Various machine learning methods have been used to establish models for classification and regression issues based on hyperspectral images. Deep learning is now a hot machine learning method with rapid development due to its nature to automatically learn the features from the data. Deep learning uses multi-layer neural networks to learn features within each layer *via* mathematical operations on input data. Deep learning can deal with big data efficiently and has been successfully applied in various fields, such as healthcare (Esteva et al., 2019), food (Zhou et al., 2019), agriculture (Kamilaris and Prenafeta-Boldú, 2018), and medical imaging (Kim et al., 2019). Deep learning has been used in hyperspectral image analysis. Due to the fact that hyperspectral image is a three-dimension (3D) data cube, one-dimension (1D), two-dimension (2D) and 3D data can be extracted from hyperspectral images. The corresponding 1D (Audebert et al., 2019; Zhang et al., 2020), 2D (Wang et al., 2018; Audebert et al., 2019), and 3D (Audebert et al., 2019; Nagasubramanian et al., 2019) deep learning models can be developed for hyperspectral image analysis. Nowadays, deep learning has been used in fruit quality and safety inspection by hyperspectral imaging, such as bruises on winter jujube (Feng et al., 2019b), strawberry ripeness (Gao et al., 2020), and early decay on blueberry (Qiao et al., 2020).

There is redundant information in hyperspectral image, which is irrelevant to the research objectives. It is important to identify which information contributes more to the research objectives. This will result in feature selection and extraction. Saliency map is a widely used visualization method for deep learning to see which information is more important for the models (Simonyan et al., 2014). Most of the studies using saliency maps focus on the classification issues. No studies have used saliency map for deep learning regression models using hyperspectral images.

In this study, hyperspectral imaging coupled with deep learning was used to identify the maturity degree of strawberry and estimate the SSC of strawberry. Deep learning models using 1D spectra and 3D hyperspectral image were developed for maturity evaluation. SSC was determined by 1D deep learning model. Saliency maps were calculated for both the classification and regression deep learning models. The performances of 1D deep learning model and 3D deep learning model for classification were also compared.

## Materials and Methods

### Sample Preparation

**Figure 1:**
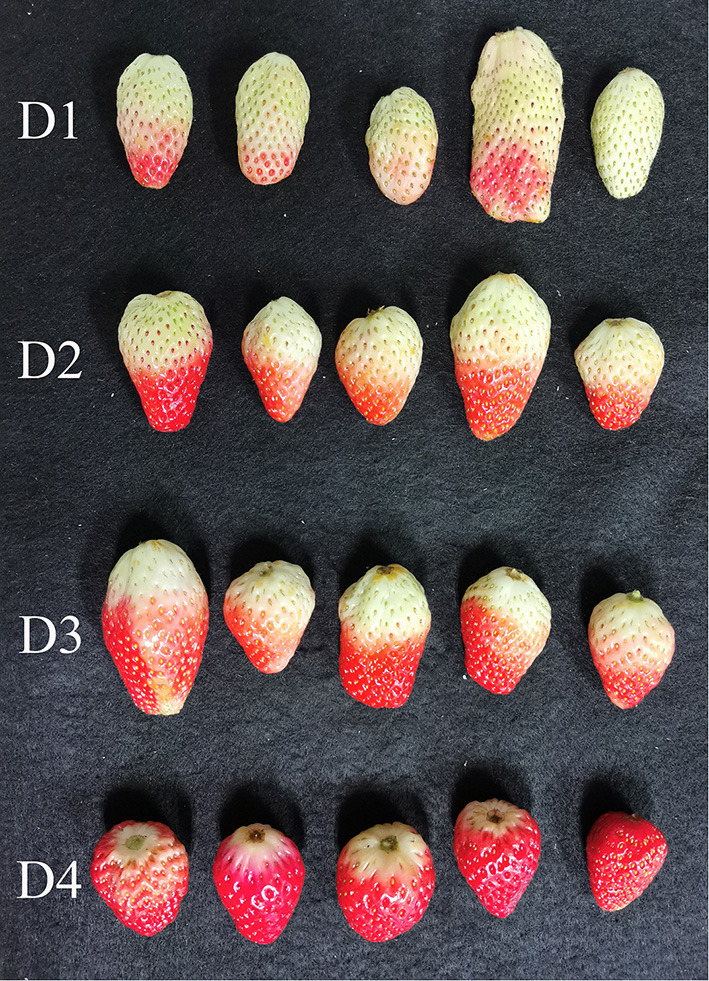
Images of strawberries of the four maturity degree (D1: the portion of the red color areas below 25%; D2: the portion of the red color areas between 26 and 50%; D3: the portion of the red color areas between 51 and 75%; D4: the portion of the red color areas between 75 and 100%).

The fresh strawberries (cultivar: Hongyan) were harvested from a local farm in Yuhang Disctrict, Hangzhou, Zhejiang Province, China in January, 2021. The strawberries were washed and cleaned. The strawberries were visually and manually divided into four maturity degrees according to the portion of the red color areas, namely Degree 1 (D1: the portion of the red color areas below 25%), Degree 2 (D2: the portion of the red color areas between 26 and 50%), Degree 3 (D3: the portion of the red color areas between 51 and 75%), and Degree 4 (D4: the portion of the red color areas between 75 and 100%). Figure 1 shows the typical samples of the four maturity degrees. After being harvested, the strawberries were cleaned and stored at room temperature. Then, hyperspectral images were acquired in the following day. For each maturity degree, 204 strawberries were collected. The samples were numbered, and 36 strawberries of each maturity degree were picked for the measurement of SSC after hyperspectral image acquisition.

### Hyperspectral Image Acquisition

A laboratory based hyperspectral imaging system covering the spectral range of 380–1,030 nm [the same in literature (Wang et al., 2020)] was used to acquire hyperspectral images of strawberries. To acquire hyperspectral images, the distance between the lens and the sample plate, the camera exposure time and the moving speed of the sample plate were adjusted as 25 cm, 0.04 s, and 3.3 mm/s. During the image acquisition procedure, these parameters were kept the same. After image acquisition, the raw hyperspectral images were corrected into the reflectance hyperspectral images (Wang et al., 2020). In each hyperspectral image, 12 strawberries were placed separately, and 17 images were acquired for each maturity degree. For strawberries of D1 and D2, the side containing more red parts were used for hyperspectral image acquisition.

### Hyperspectral Image Preprocessing and Spectra Extraction

In this study, the hyperspectral images of each strawberry were extracted from the original reflectance hyperspectral images. The head and tail of hyperspectral images contained obvious noises and only the hyperspectral images in the range of 441–947 nm (400 wavebands) were used for further analysis. A Savitzky–Golay smoothing filter was conducted on the hyperspectral images to reduce the noises (Yan et al., 2021), and area normalization was applied on the pixel-wise spectra to reduce the influence of sample shape (Zhao et al., 2020). After preprocessing, the average spectrum of each strawberry was extracted for further analysis. Both 1D spectra and the preprocessed 3D hyperspectral images were used for maturity degree classification, and 1D spectra were used for SSC evaluation.

### Determination of SSC Content

After hyperspectral image acquisition, 36 strawberries of each maturity degree were used for the SSC measurement. Each strawberry was manually squeezed with two pieces of gauze to filter the solutions. Then a drop of solution (~1 ml) was used to measure the SSC content using a portable digital refractometer instrument (30 GS, Mettler-Toledo Company, Switzerland). The refractometer was first calibrated before being used for further measurement. Table 1 summarizes the statistical analysis of the measured values of SSC of different maturity degrees.

**Table 1:** Statistical analysis of the measured SSC values of strawberries of different maturity degrees (*p* < 0.05).

**Maturity degree**	**SSC (Brix°)**
D1^*^	8.23 ± 1.15^c^
D2	8.57 ± 0.8^c^
D3	9.58 ± 1.32^b^
D4	10.37 ± 1.71^a^

* *D1: the portion of the red color areas below 25%; D2: the portion of the red color areas between 26 and 50%; D3: the portion of the red color areas between 51 and 75%; D4: the portion of the red color areas between 75 and 100%*.

*Lowercase letters (a–c) mean p < 0.05*.

As shown in Table 1, the average SSC values increased with the maturity degree of the strawberries. The SSC values of strawberries of maturity D1 and D2 did not have significant differences. While they had significant differences with D3 and D4, and D3 and D4 also had significant differences.

### Deep Learning Models

The 1D spectra and 2D images can be processed by deep learning models and conventional methods directly. However, the 3D hyperspectral image cannot be processed by the conventional methods directly. Unlike the conventional machine learning methods that may not be flexible to deal with multi-dimensional data, deep learning methods can be used to deal with multi-dimensional data effectively. Different deep learning architectures can be designed to meet the demands.

Convolutional neural network (CNN) is one of the most widely used deep learning architecture. In previous studies, the CNN models have showed equivalent or even better performances than the conventional methods for both the classification and regression. Thus, we do not use the conventional methods as a comparison in this study. After trails, the Residual Network (ResNet) architectures were used for the classification and regression using 1D spectra and 3D hyperspectral images. ResNet is a widely used CNN architecture (He et al., 2016), and it is mainly developed to deal with the degradation problem on the deeper neural networks. ResNet introduces residual blocks to solve this problem and construct very deep neural networks.

Considering the computation amount of ResNet architectures, the bottleneck block was designed as the residual block to reduce the number of parameters that have little influence on the result (Zhao et al., 2019). Since the output of the network layer close to the input was related to shallow semantics, the larger size and stride of the convolution kernel or pooling layer were set to increase the receptive field, and then, the global features were extracted. The size and stride of the network layer away from the input were set to be relatively small, so that local features were focused.

**Figure 2:**
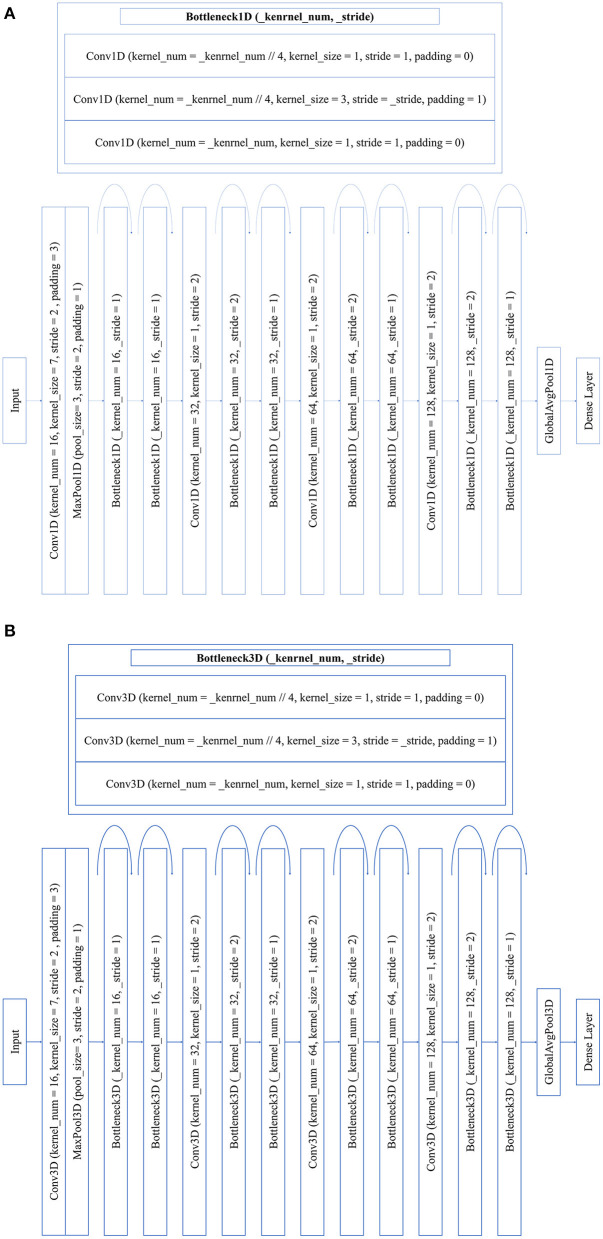
ResNet architectures for 1D spectra **(A)** and residual neural network (ResNet) architectures for 3D hyperspectral image **(B)**. The bottleneck block is defined by three convolution layers.

To avoid the inconvenience caused by different CNN architectures, we developed the same ResNet architectures for both regression and classification using 1D spectra. The differences between classification and regression lay in the loss function and dense layer. The architectures of ResNet for regression and classification of 1D spectra are shown in Figure 2A.

For 3D data, the ResNet architecture for classification is shown in Figure 2B and it is similar as the ResNet architectures for 1D spectra. We also attempted to use the 3D ResNet for the SSC determination. It failed to obtain good performances due to the high dimension data and small sample number.

For the used ResNet models, the output of each convolution layer outside the bottleneck block was batch normalized and flowed into the activation function, which was set as Rectified Linear Unit (ReLU). Notably, the input of each convolution layer in the bottleneck block was batch normalized and activated by ReLU. After global average pooling, a dropout layer was added in the dense layer, and the probability of dropout was set at 0.3. Batch normalization was applied to all datasets before training, and it was added after each convolutional layer and before the dense layer. For classification, the loss function was SoftmaxCrossEntropyLoss, the number of training epochs was 1,000, and the learning rate was 0.01. For regression, the loss function was L2 Loss, the number of training epochs was 1,500, and the learning rate was 0.0001. All the optimizers were set to Adam.

### Saliency Map

Saliency map is a visualization method in deep learning (Simonyan et al., 2014). Salience map can reflect the contribution of each data variable on the model performances. Saliency map is generally used for classification, and it is calculated using the correctly predicted samples. Generally, saliency map is used for 2D data analysis to visualize the importance of pixels for classification. It has been extended to multi-dimension data analysis for different data sources. In this study, the saliency map method proposed by Shen et al. (2018) was used, and the detailed information of saliency map used in this study for maturity degree identification can be found in Simonyan et al. (2014).

**Figure 3:**
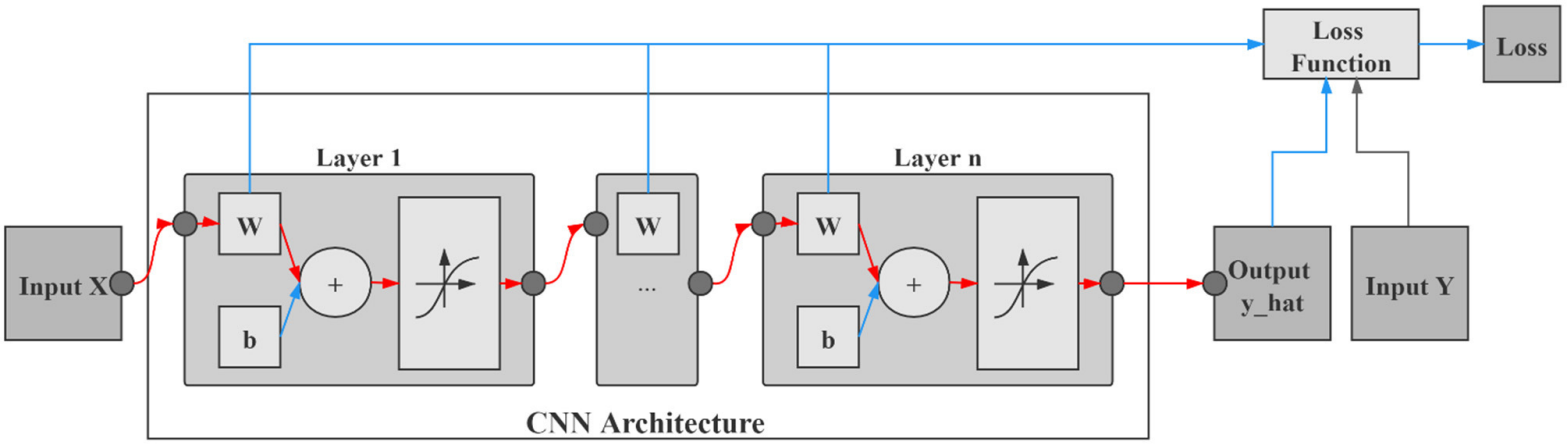
Computational graph of the propagation of convolutional neural network (CNN).

Figure 3 shows the computational graph of the propagation of CNN. The input includes sample spectral data X and label Y, the blue and red arrows represent the forward propagation direction, and the output includes the predicted values y_hat and loss. Notably, the reverse direction of the red arrow is the partial path of the CNN gradient back propagation, which is the calculation principle of saliency map. The saliency map corresponds to the dimension size of the input data and to the basic unit of the input data one by one.

In this study, the saliency map method is simply modified for regression issues to determine the SSC. As for classification issues, the saliency map is calculated using the correctly classified samples. Similarly, for regression issues, we define the “correctly predicted sample” to calculate the saliency map. The prediction error rate is used to define as the ratio of differences between the measured value and predicted value to the measured value. The samples with prediction error rate in a certain range are defined as “correctly predicted sample.” The main problem is to determine the prediction error rate. The saliency map can be calculated for each sample, and statistical analysis was conducted on each data variable of all “correctly predicted” samples to evaluate the contribution of each wavelength. In this study, L1-norm was used for the wavelength contributions. The saliency map calculation for 1D spectra and 3D hyperspectral image was conducted according to Yan et al. (2021).

### Software and Model Evaluation

Hyperspectral image of each strawberry was manually cut from the hyperspectral images (containing 12 strawberries in each image) using ENVI4.7 (ITT, Visual Information Solutions, Boulder, CO, USA). For each strawberry, hyperspectral image preprocessing and spectral extraction were conducted using Matlab R2015b (The MathWorks, Natick, MA, USA). ResNet models and saliency map were conducted on Python 3.6 using the MXNET framework (Amazon, Seattle, Washington State, USA). The performances of the classification models were evaluated by the classification accuracy, which was the ratio of the number of correctly classified samples and the number of total samples. The performances of the regression models were evaluated by the determination of coefficient (*R*^2^) and root mean square error (RMSE) of the training, validation, and testing sets.

## Results

### Spectral Profiles

**Figure 4:**
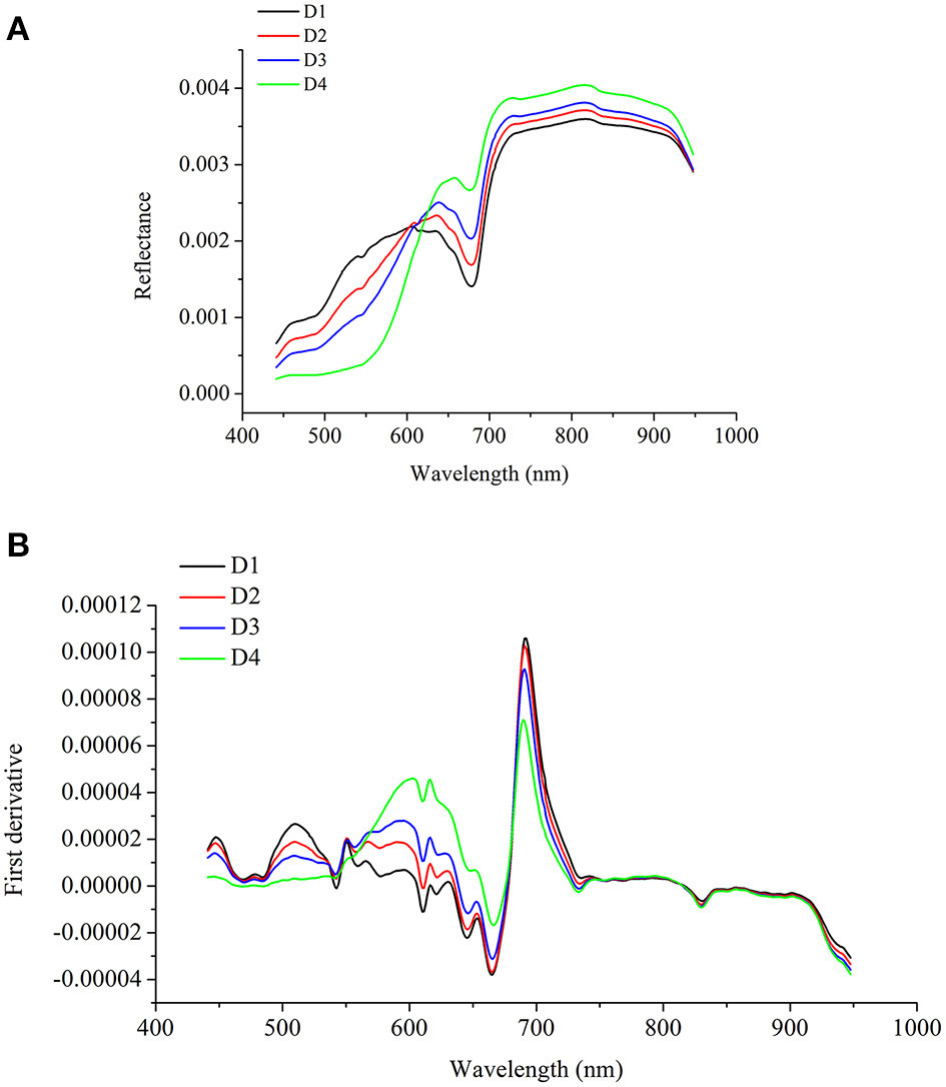
Average spectra of strawberries from different maturity degree **(A)** and corresponding first derivative spectra **(B)**.

The average spectra and the preprocessed spectra of the four maturity degrees of strawberries are shown in Figure 4, as well as the first derivative spectra of the average spectra. The average spectra and the corresponding first derivative spectra showed that there were differences in the spectral profiles of the strawberries from different maturity degrees. The main differences existed in the spectral region of 441–700 nm. The wavelength regions between 441 and 700 nm were mainly related to the color information (Tugnolo et al., 2020; Walsh et al., 2020), which has been widely used for fruit maturity degree identification, especially for the fruits with pigments changes during maturity.

### Classification of Strawberry Maturity Degree

#### Classification Results

To classify the maturity degree of strawberries, the category values of the maturity degrees were assigned as 0 (D1), 1 (D2), 2 (D3), and 3 (D4). To verify the generalization ability of these models, the samples were split into the training, validation, and testing sets for both 1D spectra and 3D hyperspectral images. In the study, 17 samples were randomly selected from each category, and each sample was successively added to the validation set and test set. The remaining samples were used as the training set. The data sampling process was repeated five times, and there was a one-to-one correspondence for each data set for the 1D spectra and 3D hyperspectral images. The number of samples in the training, validation, and testing sets was 136, 34, and 34, respectively. To establish 3D ResNet model, all hyperspectral images were resized to 85 pixels × 85 pixels × 400 wavebands.

Based on the 1D spectra and 3D hyperspectral images, ResNet models were built using the architectures shown in Figure 2. Table 2 shows the results of 1D ResNet model and 3D ResNet model. In fact, Table 2 shows the most balanced results of these models in the five repeated random data sampling process (the prediction results of these model in each data set have little fluctuation, and the results of the other four data sampling processes are shown in Supplementary Table 1. Notably, 1D ResNet model and 3D ResNet model showed the most balanced results in the same data sampling process (the samples for 1D ResNet and 3D ResNet were the same in each sampling process). It was reasonable to believe that the sample distribution of each data set was similar in this data sampling process. Good performances were obtained for the maturity degree identification. As shown in Table 2 and Supplementary Table 1, similar trends are observed for the classification of strawberry samples with different datasets. For 1D and 3D ResNet model, high classification accuracy can be found in the training, validation, and testing sets. For both 1D ResNet and 3D ResNet, the strawberries of D4 could be accurately differentiated from the other three maturity degrees. A small number of strawberries of D1 can be identified as D2. However, strawberries of D2 were more likely to be misclassified as D1 and D3, strawberries of D3 were more likely to be misclassified as D2. Indeed, the samples of each degree cover a wide range of red color percentages. The samples with close color percentages had a higher possibility to be misclassified. It should be some samples were close and belong to two different maturity degrees, which would result in misclassification.

**Table 2:** Confusion matrix of 1D and 3D ResNet model for strawberry maturity degree identification.

**Sample set**		**1D ResNet**					**3D ResNet**				
		**D1**	**D2**	**D3**	**D4**	**Accuracy**	**D1**	**D2**	**D3**	**D4**	**Accuracy**
Training	D1	131	5	0	0		135	1	0	0	
	D2	3	121	12	0		4	132	0	0	
	D3	0	8	128	0		0	8	125	3	
	D4	0	0	0	136		0	0	0	136	
	Overall					94.85%					97.06%
Validation	D1	32	2	0	0		33	1	0	0	
	D2	0	27	7	0		3	26	5	0	
	D3	0	6	27	1		1	7	23	3	
	D4	0	0	0	34		0	0	0	34	
	Overall					88.24%					85.29%
Testing	D1	29	5	0	0		33	1	0	0	
	D2	4	26	4	0		5	26	3	0	
	D3	0	5	28	1		0	8	24	2	
	D4	0	0	0	34		0	0	1	33	
	Overall					86.03%					85.29%

The classification results of 1D and 3D ResNet were quite close. The ANOVA was conducted on the classification accuracy of the three sets for the five times of modeling. No significant differences could be found between 1D and 3D ResNet models at *p* < 0.01. Although, the hyperspectral image of the strawberry provides more information than the corresponding spectrum. The reason might be that the number of samples was relatively small, the potential of deep learning for feature learning from big data might not be fully revealed.

#### Saliency Maps of 1D ResNet and 3D ResNet for Maturity Degree Identification

**Figure 5:**
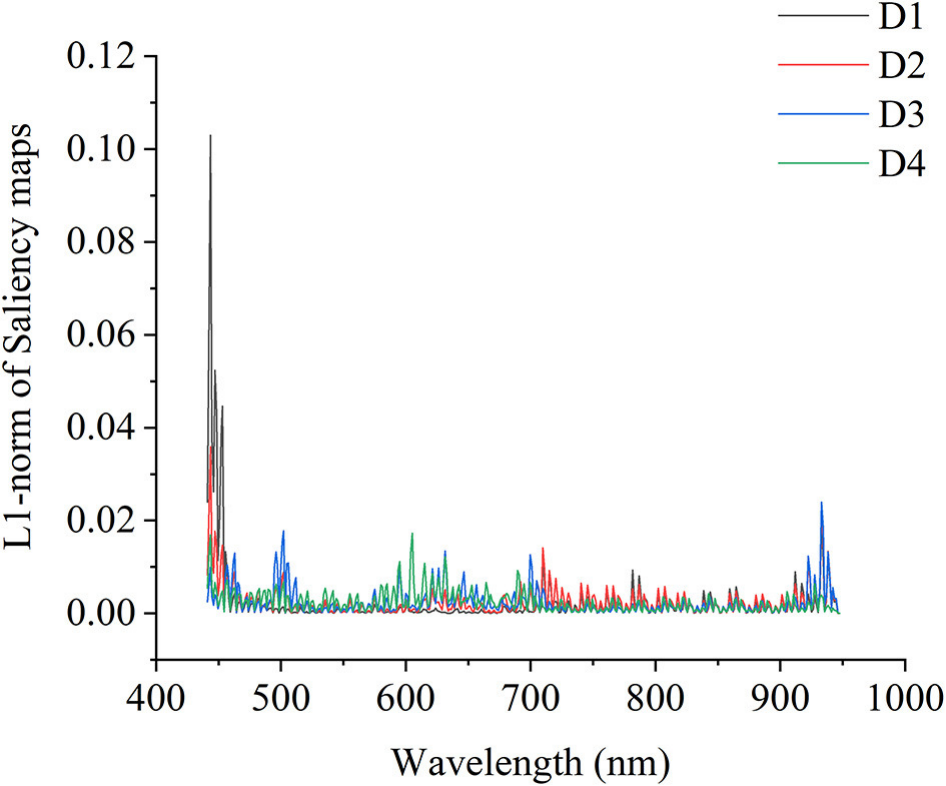
Visualization of the wavelength contribution of the testing set by 1D ResNet.

For strawberry maturity degree identification, the testing sets of 1D spectra were used for the visualization of 1D ResNet model. The models with results shown in Table 2 were used for visualization. Based on the correctly classified samples of different maturity degrees, the cumulative contribution of each wavelength was calculated, and they were normalized so that the sum of the contribution of all wavelengths was 1. Figure 5 shows the visualization of the cumulative contribution of wavelengths of samples correctly classified in the testing sets by 1D ResNet. As shown in Figure 5, the wavelengths in the ranges of 441–464, 490–513, 589–663, 688–726, and 883–947 nm showed a relatively higher contribution.

**Figure 6:**
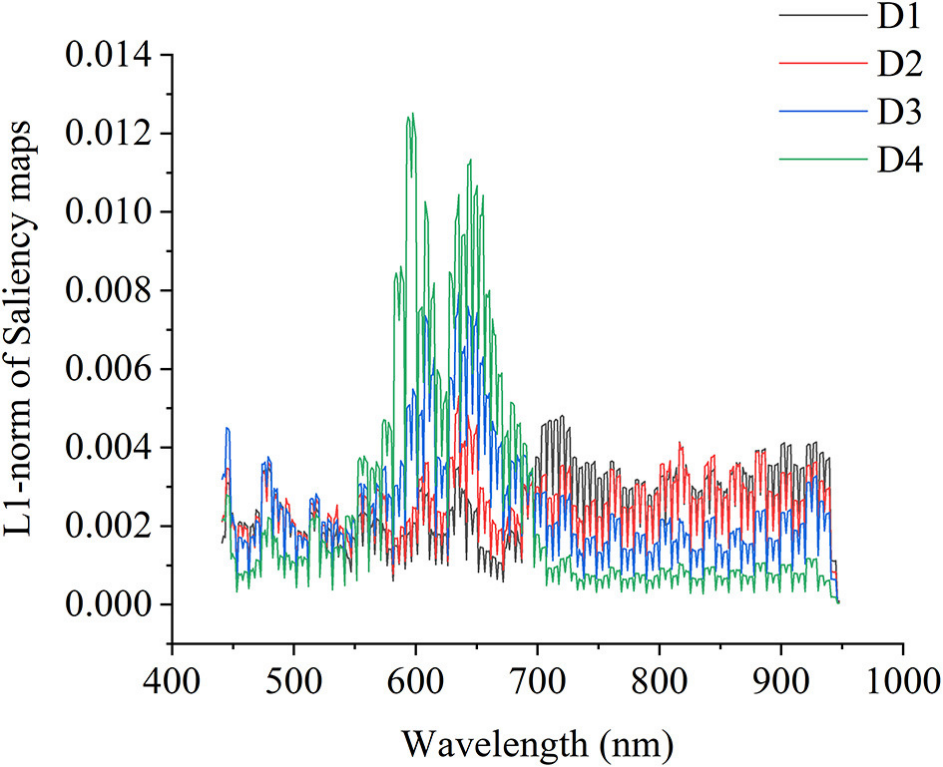
Visualization of the wavelength contribution of the testing set by 3D ResNet.

As for 3D hyperspectral image data, the visualization results of the wavelength contribution are shown in Figure 6. Based on the correctly classified samples of different maturity degrees, the cumulative contribution of each wavelength was calculated and normalized. As shown in Figure 6, in general, the 3D ResNet model was more sensitive to the spectral range of 560–680 nm. It could be noted that for each maturity degree of strawberries, the wavelength contribution showed different trends. As for D1, the wavelengths in the spectral range of 701–947 nm showed higher contributions than the spectral range of 560–680 nm. The wavelengths in the spectral range of 701–947 nm also showed a high contribution for strawberries of D1 and D2.

**Figure 7:**
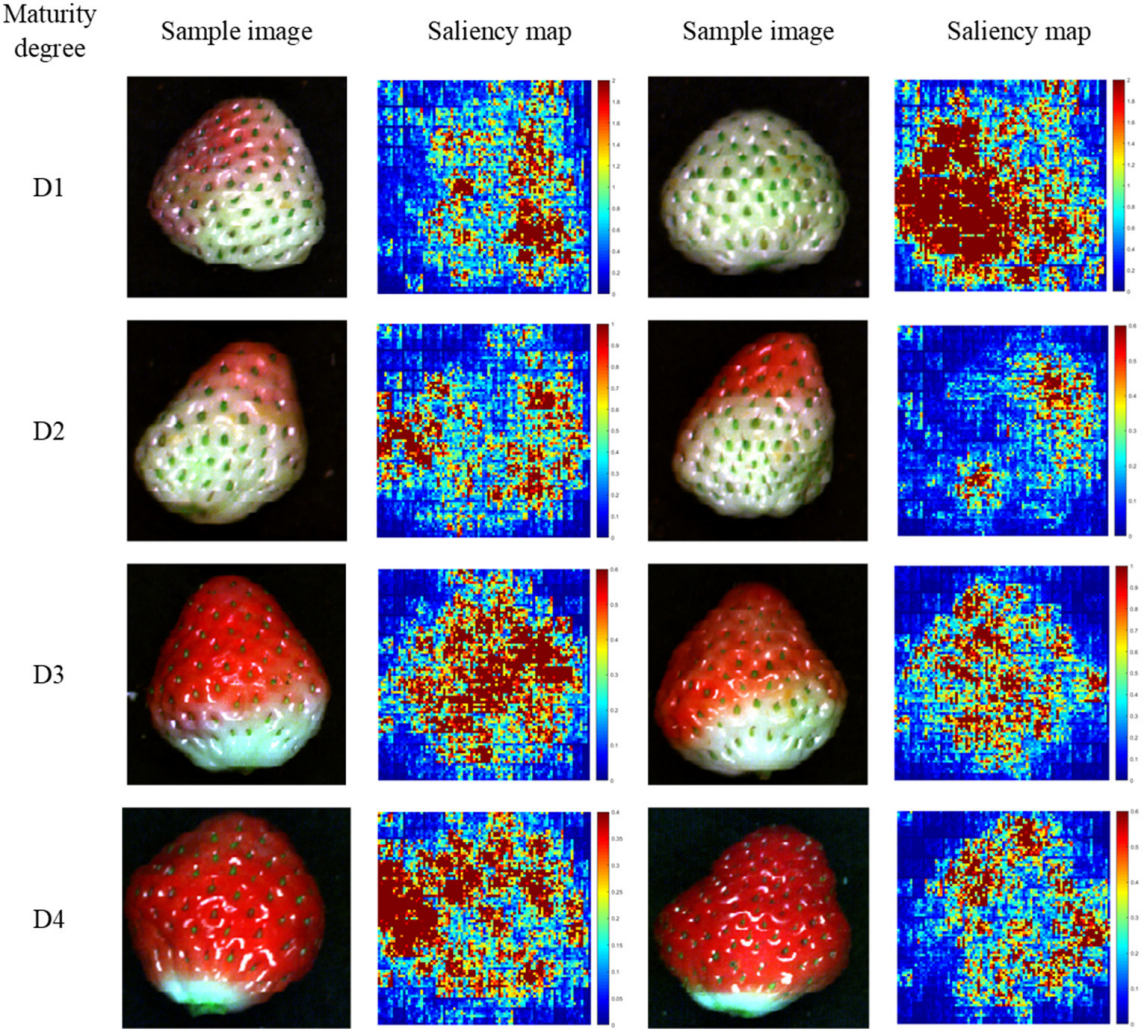
The saliency maps of two randomly selected hyperspectral images of the testing sets of each maturity degree based on 3D ResNet. Colors in the colorbar indicated the corresponding contribution values of each pixel in the hyperspectral images. The colors in the colorbar from the bottom to the top represented the values from low to high.

In 3D ResNet visualization, the visualization results of the two samples of each maturity degree are randomly presented in Figure 7. In Figure 7, the colors in the colorbar indicated the corresponding contribution values of each pixel in the hyperspectral images. The colors in the colorbar from the bottom to the top represented the values from low to high. For strawberries of D1, the green parts contributed more to the classification. For strawberries of D2, the parts with the light red color contributed more to the classification. For strawberries of D3, the red parts contributed more to the classification. For strawberries of D4, the dark red parts seemed to contribute more to the classification. As for 3D ResNet visualization, the results showed that 3D ResNet was able to learn informative features for the classification of different maturity degrees of strawberries.

### Determination of SSC

#### Results of SSC Determination

**Figure 8:**
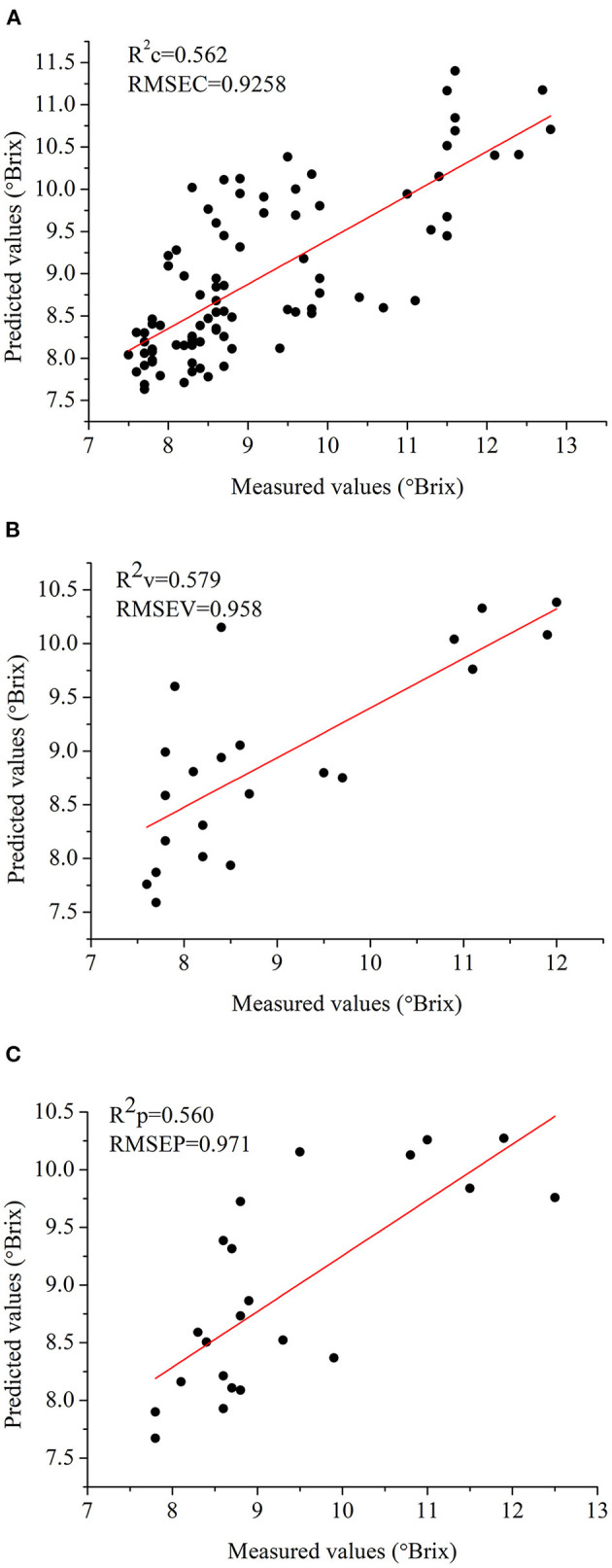
Prediction results of SSC using 1D ResNet. **(A)** Results of the training set, **(B)** results of the validation set, **(C)** results of the testing set. *R*^2^c, *R*^2^v, and *R*^2^p mean the *R*^2^ of training, validation, and testing, respectively. RMSEC, RMSEV, and RMSEP mean the root mean square error (RMSE) of training, validation and testing, respectively.

In general, 1D deep learning model was more frequently used for the purpose of evaluating the quality of food and agro-products quality. In this study, 1D ResNet model was also used to determine SSC in strawberries. Before modeling with the samples, outliers (12 samples) were removed from the sample set by a partial least square based method (He et al., 2019). Considering the spectral and biochemical properties (SSC) of the samples, SPXY method was used to maintain the appropriate distribution of samples in each data set (Wei et al., 2020). The training set, validation set, and test set were composed of 88, 22, and 22 samples, respectively. Figure 8 shows the results of the 1D ResNet for SSC determination. The results showed that the performances of 1D ResNet for SSC determination were not good enough compared with previous studies (Amodio et al., 2017; Chen et al., 2017; Shen et al., 2018; Mancini et al., 2020; Weng et al., 2020). The *R*^2^ of the three sets were all over 0.55, indicating that the improvements on the performances should be conducted in future studies. We have also tried to use 3D ResNet for SSC determination, and we failed to obtain good performances (the results were not shown) due to the high dimensionality of hyperspectral image and the small number of samples.

#### Saliency Maps of 1D ResNet for Regression

**Figure 9:**
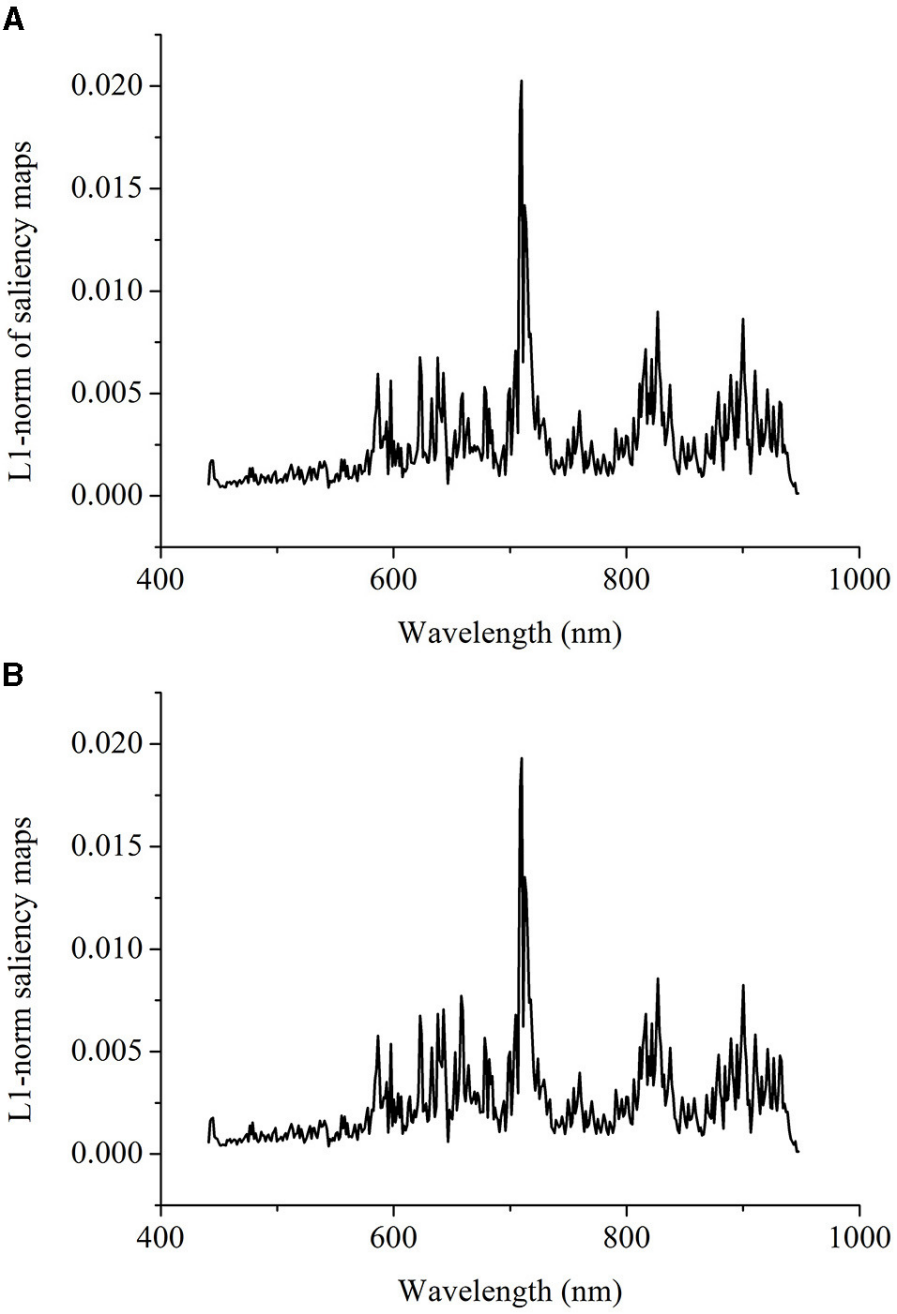
Visualization of the wavelength contribution of the testing set for the soluble solids content (SSC) determination by 1D ResNet using samples with prediction error rate of 5% **(A)** and prediction error rate of 10% **(B)**. The prediction error rate means the ratio of differences between measured value and predicted value to the measured value.

Based on the idea of the vitalization of deep learning models for classification, the saliency maps for regression issues were explored. The saliency maps for classification were calculated using the correctly classified samples. Thus, for regression issues, we defined the “correctly predicted samples” by using the prediction error rate. The prediction error rate was defined as the ratio of differences between the measured value and predicted value to the measured value. No criteria could be found for the prediction error rate. In this study, we defined the samples with a prediction error rate of 5 and 10% as “correctly predicted samples” for saliency map calculation and comparison. Figures 9A,B show the visualization of 1D ResNet model with a prediction error rate of 5 and 10%. In Figure 9A, the wavelengths in the range of 700–720 nm showed higher contributions, followed by the wavelengths in the range of 810–840, 880–940, and 580–600 nm. In Figure 9B, similar results as Figure 9A could be found for the prediction error rate of 10%. Some of the wavelengths in these regions could be found in literature (Choi et al., 2017; Li et al., 2020) for the SSC determination.

## Discussion

Fruit maturity is a concerned issue for planter, trader, and consumers. Hyperspectral imaging has been proved to be an effective technique for fruit maturity and quality evaluation. In this study, good performances were obtained for strawberry maturity degree evaluation, and the evaluation of SSC values was not so good.

As for fruit maturity evaluation by hyperspectral imaging, spectral features (Zhang et al., 2016; Shao et al., 2020), image features (Elmasry et al., 2007; Zhang et al., 2016; Gao et al., 2020), and the fusion of these features were used as inputs of classification models (Zhang et al., 2016; Khodabakhshian and Emadi, 2017). The spectral features were the most widely used. The fusion of spectral features and image features from hyperspectral images generally obtained good performances (Zhang et al., 2016; Khodabakhshian and Emadi, 2017). In this study, in addition to the spectra of the 1D ResNet, the hyperspectral images were used as inputs of 3D ResNet, without extracting the spectral features and image features separately in advance. As for maturity evaluation, good performances were obtained for both 1D spectra and 3D hyperspectral image. Better training performances were obtained by 3D ResNet, while worse validation and testing performances were obtained by 3D ResNet. The results indicated that both 1D CNN and 3D CNN could be used to identify the maturity degree of strawberries. The ANOVA of the classification accuracy of the three sets for the five times of modeling showed that there were no significant differences (p<0.01) on the performances between 1D and 3D ResNet models. Indeed, the training of 3D CNN took much more time and computation cost than that of 1D CNN, and the prediction using 3D CNN was fast (~0.0156 s in this study).

As for hyperspectral image for fruit SSC determination, the spectral features, image features, and the fusion of spectral features and image features have also been used (Fan et al., 2016; Weng et al., 2020; Huang et al., 2021; Pang et al., 2021). In addition to 1D spectra of 1D ResNet, 3D hyperspectral images with more spectral and image features could be used to establish 3D ResNet model, without the use of pre-extracted spectral features and image features. After trials in this study, we failed to use 3D ResNet for the SSC determination, due to the fact that the dimension of hyperspectral image was high and the number of the used samples was small. Indeed, the prediction performances of SSC were not good enough. In some studies, the results of SSC determination using visible/near infrared spectroscopy and hyperspectral imaging were also not good enough (Leiva-Valenzuela et al., 2014; Pu et al., 2015; Li et al., 2016; Anisur et al., 2017; Ekramirad et al., 2017). Indeed, our results for the SSC determination of strawberries were not as good as those for strawberries SSC determination using visible/near-infrared spectroscopy and hyperspectral imaging (Amodio et al., 2017; Chen et al., 2017; Shen et al., 2018; Mancini et al., 2020; Weng et al., 2020). In all, the prediction results of fruit SSC varied, and more efforts were needed to improve the performances of the SSC determination of strawberries based on this study.

This study illustrated the feasibility of deep learning approaches for strawberry maturity and quality evaluation. For maturity evaluation, the computation cost of 3D ResNet was significantly more than that of 1D ResNet. Although hyperspectral images contained more information, the potential of 3D ResNet for feature learning from the hyperspectral images might have not been fully revealed with the small number of samples. On the other hand, only one variety of strawberries was studied. It was important to develop models for the different varieties of strawberry in the future.

Beyond the modeling procedures, the saliency map method was used to visualize the important information relating to the classification and regression. By using saliency map methods, the important spectral regions and important pixels in the hyperspectral image for maturity and SSC evaluation could be explored. For maturity identification, the wavelength contribution of 1D and 3D ResNet showed differences. The wavelengths with higher contribution matched with the wavelengths with larger differences in the first derivative spectra. The wavelengths in the range of 589–663 nm contributed more for both 1D and 3D ResNet. All these wavelengths were related to the pigments (color) information (Tugnolo et al., 2020; Walsh et al., 2020). Moreover, for 3D ResNet visualization in Figure 7, it could be found that the pixels with higher contributions were related to the parts with different color information.

The saliency map methods are generally used for classification issues. However, based on its principles, it might be able to extend the saliency map to the regression issues. In this study, the saliency maps of 1D ResNet estimation were calculated. The wavelengths that contributed more to the SSC determination lay in the spectral regions of 700–720, 810–840, 880–940, and 580–600 nm. The saliency maps for the regression could be conducted. The main problem was that samples could be used to calculate the saliency maps. In this study, we used the prediction error rate to identify the “correctly predicted samples.” More efforts should be conducted to extend the saliency map and similar methods to the regression issues.

## Conclusion

Hyperspectral imaging coupled with deep learning approaches were used to classify strawberries from different maturity degrees and estimate the SSC in strawberries. Both 1D spectra and 3D hyperspectral images were used to establish the ResNet models for maturity degree identification, and 1D spectra were used for the SSC estimation. Good performances were obtained for maturity degree identification, with the classification accuracy over 90% in the training set and classification accuracy over 84% in the validation and testing sets for both 1D and 3D ResNet. For SSC determination, the performances of 1D ResNet were not good enough, with *R*^2^c, *R*^2^v, and *R*^2^p ~ 0.55. For classification, 1D and 3D ResNet showed close results, and the computation cost of 3D ResNet was more than that of 1D ResNet. The results indicated that 1D spectra were able to identify strawberry maturity degree and determine SSC with a small number of samples. With more samples, the potential of 3D ResNet might be fully revealed due to the feature learning abilities of deep learning in big data analysis. The saliency maps of wavelengths and pixels in 1D and 3D ResNet showed that the pigments related information (color information) contributed more for maturity degree identification in strawberry using the hyperspectral imaging. The saliency maps of 1D ResNet for the SSC determination were also explored, and the wavelengths in the spectral range of 700–720, 810–840, 880–940, and 580–600 nm contributed more for the SSC determination in this study. The overall results showed that the hyperspectral imaging combined with deep learning approaches could be used to identify maturity degree and predict SSC of strawberry. More efforts should be made to improve the performances of SSC prediction. The 3D deep learning models could be used for the hyperspectral image analysis of fruits and it could be extended to other food and agro-products quality inspection, although it did not outperform the 1D deep learning models with a small number of samples. The use of 3D deep learning models using hyperspectral images for regression should be further investigated with more samples and effective dimensionality reduction algorithms.

## Data Availability Statement

The raw data supporting the conclusions of this article will be made available by the authors, without undue reservation.

## Author Contributions

ZS, CZ, and LFe: conceptualization, formal analysis, and project administration. CZ, TY, and ZS: data curation and writing—original draft. LFe and ZS: funding acquisition, supervision, and writing—review and editing. ZS: investigation. JZ, YZ, XL, TY, PG, and CZ: methodology. JZ, YZ, XL, ZS, LH, and LFa: resources. CZ, TY, PG, and LFa: software. CZ and TY: validation. ZS and CZ: visualization. All authors contributed to the article and approved the submitted version.

## Funding

This work was supported by Six-Party Science and Technology Cooperation Project of Zhejiang Province (CTZB-F160728AWZ-SNY1-10), Public Technology Research Program of Zhejiang Province (LGN20C030002), National Natural Science Foundation of China (31871526), and XPCC Science and Technology Projects of Key Areas (2020AB005).

## Conflict of Interest

LH and LFa were employed by the company Hangzhou Liangzhu Linhai Vegetable and Fruit Professional Cooperative. The remaining authors declare that the research was conducted in the absence of any commercial or financial relationships that could be construed as a potential conflict of interest.

## Publisher's Note

All claims expressed in this article are solely those of the authors and do not necessarily represent those of their affiliated organizations, or those of the publisher, the editors and the reviewers. Any product that may be evaluated in this article, or claim that may be made by its manufacturer, is not guaranteed or endorsed by the publisher.
