# Supplementary material for: Application of Hyperspectral Imaging for Maturity and Soluble Solids Content Determination of Strawberry With Deep Learning Approaches
Source: Front Plant Sci. 2021 Sep 10;12:736334. doi: 10.3389/fpls.2021.736334 (PMC8462090; doi:10.3389/fpls.2021.736334)
Supplement: Supplementary file 1 [file Table_1.DOCX]

Supplementary Material

**Supplementary Table 1. Confusion matrix of 1D ResNet model and 3D ResNet model for strawberry maturity degree identification with other four sampling processes.**

| Sampling frequency | Sample set |  | 1D ResNet | | | | | 3D ResNet | | | | |
| --- | --- | --- | --- | --- | --- | --- | --- | --- | --- | --- | --- | --- |
|  |  |  | D1 | D2 | D3 | D4 | accuracy | D1 | D2 | D3 | D4 | accuracy |
| 1 | Training | D1 | 130 | 6 | 0 | 0 |  | 126 | 10 | 0 | 0 |  |
|  |  | D2 | 6 | 114 | 16 | 0 |  | 4 | 131 | 0 | 0 |  |
|  |  | D3 | 0 | 13 | 121 | 2 |  | 0 | 42 | 87 | 7 |  |
|  |  | D4 | 0 | 0 | 0 | 136 |  | 0 | 0 | 2 | 134 |  |
|  |  | overall |  |  |  |  | 92.10% |  |  |  |  | 87.87% |
|  | Validation | D1 | 31 | 3 | 0 | 0 |  | 32 | 2 | 0 | 0 |  |
|  |  | D2 | 2 | 30 | 2 | 0 |  | 2 | 32 | 0 | 0 |  |
|  |  | D3 | 0 | 7 | 27 | 0 |  | 0 | 17 | 17 | 0 |  |
|  |  | D4 | 0 | 0 | 0 | 34 |  | 0 |  | 1 | 33 |  |
|  |  | overall |  |  |  |  | 89.71% |  |  |  |  | 83.82% |
|  | Testing | D1 | 32 | 2 | 0 | 0 |  | 28 | 6 | 0 | 0 |  |
|  |  | D2 | 2 | 25 | 0 | 0 |  | 1 | 33 | 0 | 0 |  |
|  |  | D3 | 0 | 3 | 30 | 1 |  | 0 | 9 | 24 | 1 |  |
|  |  | D4 | 0 | 0 | 1 | 33 |  | 0 | 0 | 3 | 31 |  |
|  |  | overall |  |  |  |  | 88.24% |  |  |  |  | 85.29% |
| 2 | Training | D1 | 128 | 8 | 0 | 0 |  | 136 | 0 | 0 | 0 |  |
|  |  | D2 | 8 | 111 | 17 | 0 |  | 0 | 136 | 0 | 0 |  |
|  |  | D3 | 0 | 14 | 122 | 7 |  | 0 | 0 | 136 | 0 |  |
|  |  | D4 | 0 | 0 | 5 | 131 |  | 0 | 0 | 0 | 136 |  |
|  |  | overall |  |  |  |  | 90.44% |  |  |  |  | 100.00% |
|  | Validation | D1 | 30 | 4 | 0 | 0 |  | 31 | 3 | 0 | 0 |  |
|  |  | D2 | 1 | 30 | 3 | 0 |  | 3 | 29 | 2 | 0 |  |
|  |  | D3 | 0 | 2 | 31 | 1 |  | 0 | 2 | 30 | 2 |  |
|  |  | D4 | 0 | 0 | 3 | 31 |  | 0 | 0 | 1 | 33 |  |
|  |  | overall |  |  |  |  | 89.71% |  |  |  |  | 90.44% |
|  | Testing | D1 | 31 | 3 | 0 | 0 |  | 30 | 4 | 0 | 0 |  |
|  |  | D2 | 3 | 27 | 4 | 0 |  | 2 | 26 | 6 | 0 |  |
|  |  | D3 | 0 | 9 | 24 | 1 |  | 0 | 8 | 25 | 1 |  |
|  |  | D4 | 0 | 0 | 1 | 33 |  | 0 | 0 | 1 | 33 |  |
|  |  | overall |  |  |  |  | 84.56% |  |  |  |  | 83.82% |
| 3 | Training | D1 | 133 | 3 | 0 | 0 |  | 136 | 0 | 0 | 0 |  |
|  |  | D2 | 10 | 98 | 28 | 0 |  | 24 | 106 | 6 | 0 |  |
|  |  | D3 | 0 | 17 | 118 | 1 |  | 1 | 31 | 97 | 7 |  |
|  |  | D4 | 0 | 0 | 3 | 133 |  | 0 | 0 | 0 | 136 |  |
|  |  | overall |  |  |  |  | 88.60% |  |  |  |  | 87.32% |
|  | Validation | D1 | 30 | 4 | 0 | 0 |  | 32 | 2 | 0 | 0 |  |
|  |  | D2 | 2 | 27 | 5 | 0 |  | 4 | 30 | 0 | 0 |  |
|  |  | D3 | 0 | 3 | 30 | 1 |  | 0 | 7 | 25 | 2 |  |
|  |  | D4 | 0 | 0 | 0 | 34 |  | 0 | 0 | 0 | 34 |  |
|  |  | overall |  |  |  |  | 88.97% |  |  |  |  | 88.97% |
|  | Testing | D1 | 33 | 1 | 0 | 0 |  | 34 | 0 | 0 | 0 |  |
|  |  | D2 | 2 | 29 | 3 | 0 |  | 7 | 25 | 2 | 0 |  |
|  |  | D3 | 0 | 6 | 26 | 2 |  | 0 | 6 | 25 | 3 |  |
|  |  | D4 | 0 | 0 | 0 | 34 |  | 0 | 0 | 1 | 33 |  |
|  |  | overall |  |  |  |  | 89.71% |  |  |  |  | 86.03% |
| 4 | Training | D1 | 126 | 10 | 0 | 0 |  | 134 | 2 | 0 | 0 |  |
|  |  | D2 | 5 | 106 | 25 | 0 |  | 22 | 109 | 5 | 0 |  |
|  |  | D3 | 0 | 18 | 117 | 1 |  | 2 | 28 | 101 | 5 |  |
|  |  | D4 | 0 | 0 | 0 | 136 |  | 0 | 0 | 0 | 136 |  |
|  |  | overall |  |  |  |  | 89.15% |  |  |  |  | 88.24% |
|  | Validation | D1 | 30 | 4 | 0 | 0 |  | 33 | 1 | 0 | 0 |  |
|  |  | D2 | 0 | 33 | 1 | 0 |  | 5 | 26 | 3 | 0 |  |
|  |  | D3 | 0 | 4 | 28 | 2 |  | 0 | 6 | 25 | 3 |  |
|  |  | D4 | 0 | 0 | 0 | 34 |  | 0 | 0 | 0 | 34 |  |
|  |  | overall |  |  |  |  | 91.91% |  |  |  |  | 86.76% |
|  | Testing | D1 | 33 | 1 | 0 | 0 |  | 34 | 0 | 0 | 0 |  |
|  |  | D2 | 2 | 27 | 5 | 0 |  | 8 | 24 | 2 | 0 |  |
|  |  | D3 | 0 | 3 | 30 | 1 |  | 1 | 5 | 23 | 5 |  |
|  |  | D4 | 0 | 0 | 3 | 31 |  | 0 | 0 | 0 | 34 |  |
|  |  | overall |  |  |  |  | 88.97% |  |  |  |  | 84.56% |
